# The intersection of chemokine signaling with the hallmarks of cancer in glioblastoma

**DOI:** 10.1007/s11060-026-05768-0

**Published:** 2026-08-26

**Authors:** Rafal Chojak, Jillyn R Turunen, Noah B Drewes, Lara Koutah, Jawad Fares, Rebecca X Chen, Ruochen Du, Umme H Faisal, Hasaan A Kazi, Clare Kelley, Jason Miska, Amy B Heimberger, Atique U Ahmed, Maciej S. Lesniak

**Affiliations:** 1 Department of Neurological Surgery, Feinberg School of Medicine, Northwestern University, 676 N. St. Clair St., Suite 2210, Chicago, IL 60611 USA; 2 Malnati Brain Tumor Institute of the Robert H. Lurie Comprehensive Cancer Center, Feinberg School of Medicine, Northwestern University, Chicago, IL 60611 USA

**Keywords:** Glioblastoma, Chemokines, Tumor microenvironment, Cancer hallmarks, Therapeutic resistance, Immunotherapy

## Abstract

**Purpose:**

Glioblastoma, IDH-wildtype, is characterized by diffuse invasion, profound immunosuppression, treatment resistance, and near-inevitable recurrence. Although canonical genetic alterations establish malignant capacity, they do not fully explain how glioblastoma cells adapt to hypoxic, perivascular, invasive, immunosuppressive, metabolically constrained, and treatment-injured microenvironments. We examined how chemokine signaling contributes to these adaptive behaviors.

**Methods:**

We used the hallmarks of cancer as an organizing framework to synthesize preclinical, translational, and clinical evidence on chemokine circuits in glioblastoma. We evaluated recurrent mechanistic pathways, distinguished causal functions from context-dependent biomarker associations, and assessed their therapeutic relevance.

**Results:**

Chemokines act primarily as spatial and stress-responsive regulators rather than initiating oncogenic drivers. Recurrent circuits include CXCL12–CXCR4 in vascular repair, invasion, and stem-like persistence; CCL2 and CCL7 signaling through CCR2 in suppressive myeloid recruitment and metabolic–immune remodeling; CCL5–CCR5 in perivascular protection, invasion, and DNA-damage tolerance; and CXCL8 signaling through CXCR1 and CXCR2 in angiogenesis, immune evasion, and therapy-induced plasticity. Most chemokine-directed strategies remain preclinical or early translational.

**Conclusion:**

Therapeutic development should prioritize biomarker-defined dependencies and appropriately timed combinations that disrupt selected chemokine-dependent interactions within specific biological and treatment contexts. Clinical translation will require verification of target engagement, disruption of the relevant cellular interactions, and evidence that chemokine modulation improves treatment response or delays recurrence.

## Introduction

Glioblastoma, IDH-wildtype, CNS WHO grade 4, is an adult-type diffuse glioma driven by recurrent alterations in growth-factor signaling, cell-cycle and p53 control, telomere maintenance, and DNA-damage responses [1–12]. These lesions establish malignant competence but do not fully explain diffuse invasion, treatment resistance, or near-inevitable recurrence [13–20]. Those behaviors also depend on hypoxic, perivascular, infiltrative, immunosuppressive, and therapy-injured microenvironments that promote adaptive and therapy-tolerant states [13–31].

Chemokines are small (approximately 8–12 kDa) secreted cytokines that signal mainly through G protein–coupled receptors [21–25, 27–29, 32, 33]. By forming spatial gradients, they direct cell migration, tissue positioning, and intercellular interactions. These gradients are shaped by regulated chemokine production, proteolytic processing, binding to extracellular-matrix glycosaminoglycans, and scavenging by atypical chemokine receptors, which together determine ligand availability, stability, and spatial range [34–36]. Although classically associated with leukocyte trafficking [37, 38], chemokines in glioblastoma are produced and sensed across malignant, myeloid, glial, endothelial, and perivascular compartments [21, 25, 29, 32, 33]. As such, chemokine gradients can regulate invasion [23–25], vascular remodeling and repair [27], stem-like persistence [22, 28, 33], and recovery after radiotherapy or temozolomide (TMZ) [24, 27–29]. Current evidence generally positions chemokines not as initiating factors, but as microenvironmental amplifiers that localize, reinforce, and stabilize stress-adaptive programs [1–3, 21–23, 26, 31, 32, 39, 40].

**Fig. 1:**
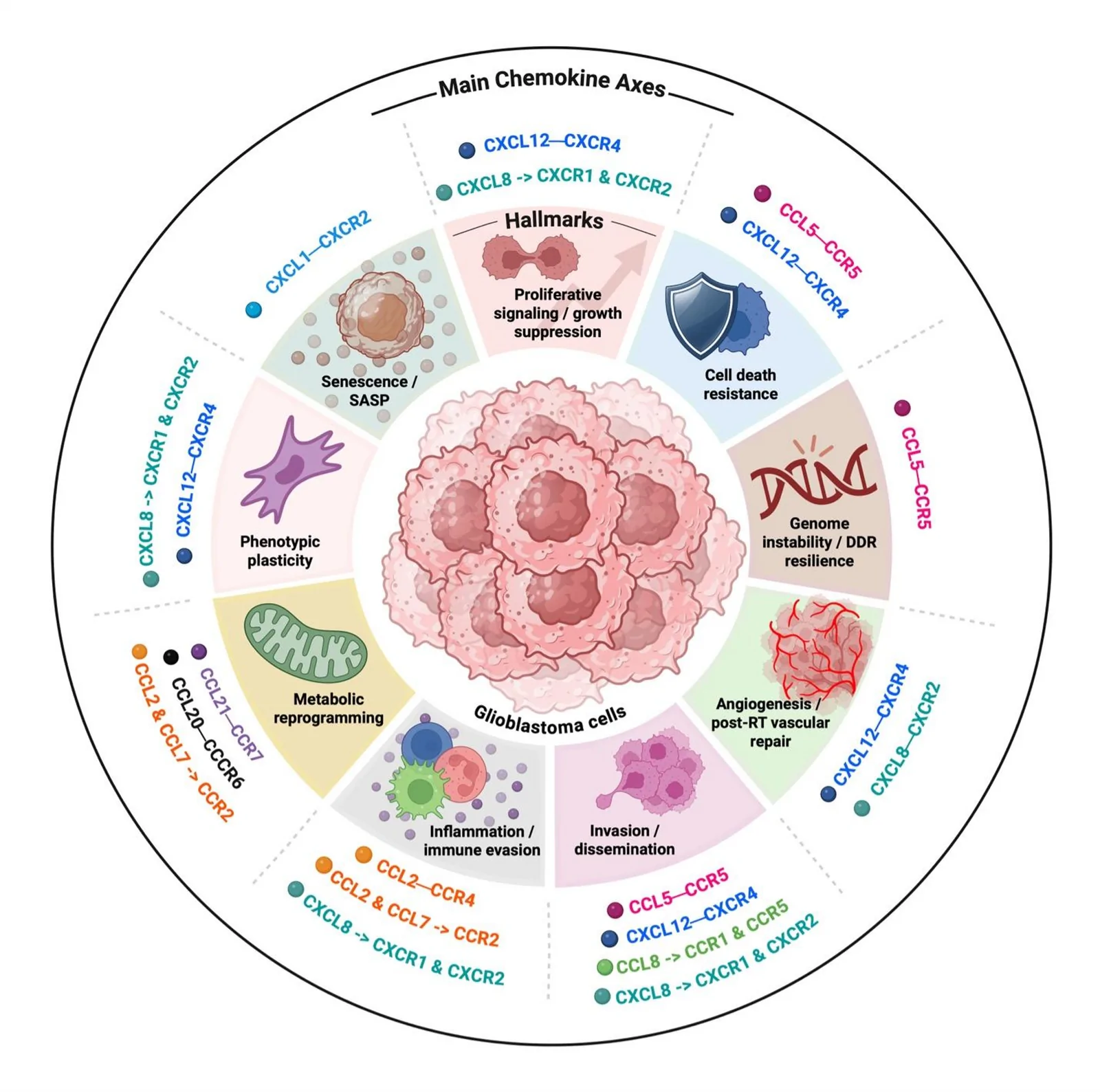
Overview of chemokine signaling across glioblastoma hallmarks. Major chemokine circuits are mapped to the hallmarks in which they have direct mechanistic or context-dependent roles, highlighting recurrent pathways that contribute to multiple malignant phenotypes. DDR, DNA-damage response; SASP, senescence-associated secretory phenotype

Despite expanding evidence, chemokine biology in glioblastoma remains conceptually fragmented, with studies often focused on individual axes, cellular compartments, or treatment contexts rather than integrated disease mechanisms. The key challenge is distinguishing mechanistic chemokine drivers from context-dependent markers of niche composition or treatment-induced stress [21, 27, 40]. Accordingly, this review maps chemokine signaling onto the major cancer hallmarks [41, 42] in glioblastoma by defining major circuits that shape invasion, immune suppression, cellular plasticity, vascular adaptation, therapy resistance, and recurrence. Because the inferred function of a chemokine circuit depends on model context, we identify whether evidence derives from established cell lines, patient-derived cultures or xenografts, immunocompetent syngeneic gliomas, genetically engineered models, or human tissue. We distinguish biomarkers from therapeutic targets and experimentally plausible interventions from clinically actionable strategies. Organized through this hallmark framework, chemokine signaling can clarify mechanisms of glioblastoma progression, expose targetable vulnerabilities, and support rational combination strategies. The major chemokine circuits discussed in this review and their relationships to individual glioblastoma hallmarks are summarized in Fig. 1.

### Sustaining proliferative signaling and evading growth suppression

In glioblastoma, sustained proliferation and evasion of growth restraint are driven primarily through alterations in the RTK–RAS–PI3K axis, the CDKN2A/B–RB cell-cycle checkpoint, the p53 pathway, and PTEN-dependent PI3K restraint [1–3]. EGFR amplification and EGFRvIII are frequent alterations within this circuitry [1–3], but their spatial heterogeneity, subclonality, and evolutionary plasticity limit their ability to define a uniform, druggable dependency across the tumor mass [1–3, 43–50]. Available evidence places chemokines downstream of, or parallel to, these canonical lesions rather than as substitutes for them [1–3, 23, 32, 51–54]. Their best-supported role is to lower the threshold for proliferation or preserve cycling in tumor cells whose intrinsic growth restraints are already compromised [23, 32, 51–54].

CXCL12–CXCR4 is one of the best-supported preclinical examples of this principle [23, 33, 55–59]. CXCL12, also known as stromal cell-derived factor-1 (SDF-1), has context-dependent malignant and stromal sources, including glioblastoma cells, SVZ astrocytes, and endothelial cells within vascular and invasive niches [52, 59, 60]. CXCL12 can stimulate glioblastoma-cell proliferation and motility through ERK and AKT signaling in experimental systems [32, 51, 55, 61, 62]. In a hypoxic neural-progenitor-derived glioblastoma model, tumor cells produced CXCL12 and expressed CXCR4, forming an autocrine loop that supported proliferation under oxygen stress [52].

CXCL8 (also known as IL-8), acting through CXCR1 and CXCR2, provides a second chemokine-linked growth-support axis [22, 28, 53, 54]. In a three-dimensional coculture and subcutaneous SCID xenograft model, endothelial-derived CXCL8 promoted growth and migration of patient-derived glioblastoma stem-like cells, providing mechanistic rather than orthotopic validation [22]. After TMZ exposure, glioblastoma cells themselves upregulated CXCL8, establishing an autocrine CXCL8–CXCR2 loop that enriched tumor-initiating states in vitro and in intracranial xenografts [28]. PTEN-deficient glioblastoma cells also showed increased CXCL8 transcription after loss of STAT3-dependent repression [53, 54].

The use of chemokine blockade as a therapeutic strategy to suppress glioblastoma proliferation remains largely preclinical [61]. For example, the CXCR4 antagonist plerixafor (AMD3100) reduces intracranial brain-tumor growth in preclinical models, in part by decreasing proliferation and ERK/AKT activation [61]. Clinical studies show that plerixafor can reach both cerebrospinal fluid and resected tumor tissue [63]. However, drug detection alone does not establish receptor occupancy, pathway suppression, or biologically active exposure across spatially distinct glioblastoma niches [63–66]. Clinical development around this hallmark has therefore focused mainly on canonical proliferative drivers rather than chemokine circuits.

### Resisting cell death and enabling replicative immortality

Cell-death resistance and replicative immortality are related but mechanistically distinct hallmarks in glioblastoma [1, 3, 18, 19, 29, 52]. Chemokine signaling is linked much more strongly to therapy-tolerant survival than to telomere maintenance [18, 19, 29, 67]. Tumor-intrinsic resistance to lethal stress is shaped by p53-pathway disruption, PI3K/AKT survival signaling, checkpoint defects, and DNA-repair capacity, whereas CDKN2A/B–RB–CDK4/6 alterations primarily disable senescence and growth arrest [1, 3, 8, 9, 18, 19]. In IDH-wildtype glioblastoma, replicative immortality is most commonly secured through TERT promoter mutation, which creates de novo transcription-factor binding sites and increases TERT expression [68–70].

The perivascular niche provides a clear example of chemokine-supported survival. Pericytes can secrete CCL5, which activates CCR5 on adjacent glioblastoma cells and protects them from TMZ-induced cytotoxicity [29]. Reactive oligodendrocytes provide an additional stromal source of CCL5, supporting the growth and stemness of CCR5-expressing glioma stem-like cells [71].

CXCL12–CXCR4 has also been studied in an SVZ-homing xenograft model in which patient-derived GB138 glioblastoma cells were implanted into the striatum of immunodeficient mice; a subset migrated along the *corpus callosum* to the host adult SVZ [59, 67]. This is a model of tumor-cell homing to the SVZ, not a tumor arising from the SVZ. In this model, CXCL12 released by the host SVZ - localized in the preceding invasion study to SVZ astrocytes and associated vasculature - promoted mesenchymal-like features and relative radioresistance in SVZ-nested tumor cells, whereas CXCR4 blockade attenuated these features and increased radiosensitivity [59, 67]. CXCL8 could also align with this hallmark only under a narrower interpretation. Rather than acting as a direct, cell-autonomous apoptosis blocker across glioblastoma cells, CXCL8 signaling through CXCR1 and CXCR2 appears to preserve or regenerate stem-like, tumor-initiating populations after niche or therapy stress [22, 28].

Replicative immortality in glioblastoma is primarily a genetically encoded capability rather than a chemokine-driven phenotype [4, 5, 68–70]. Although hypoxia, NF-κB activity, and inflammatory cytokine programs can modulate TERT expression in some contexts [72–74], a direct chemokine-to-telomere-maintenance mechanism in glioblastoma remains unproven.

Current therapies define the cytotoxic and mitotic pressures that chemokine-rich niches may buffer, but they do not validate chemokine-directed treatment. Radiotherapy/TMZ, lomustine/TMZ in selected MGMT-methylated disease, and tumor-treating fields improve outcomes through DNA-damaging or antimitotic mechanisms [6, 7, 75–77]. Their relevance here is therefore contextual: they create the treatment injury against which perivascular CCL5–CCR5 and CXCL12–CXCR4 survival circuits may operate [29, 52, 67].

Clinical translation of chemokine-survival blockade in glioblastoma remains preliminary [29, 63, 78]. Although maraviroc and plerixafor are clinically available CCR5 and CXCR4 antagonists, respectively, their use in glioblastoma remains investigational. Maraviroc has been tested in preclinical glioblastoma models. Current evidence supports pharmacologic feasibility rather than clinical validation [29, 63, 78, 79].

### Genome instability and DNA-damage resilience

Genome instability provides the substrate for glioblastoma evolution. DNA-damage responses then determine whether therapy-injured cells die, repair damage, enter persistent stress-adapted states, become immunologically visible, or survive to seed recurrence [1, 3, 7–9, 19, 75, 80–83]. Chemokines are not primary mutagens in glioblastoma, nor do they replace the tumor-cell-intrinsic genomic programs. Their more specific role is to shape the fate of genotoxically injured cells. For example, chemokines can promote DNA-damage tolerance and repair, whereas unresolved DNA damage can induce chemokine programs that alter immune visibility [27, 29, 67, 78, 84–89].

Standard radiotherapy and temozolomide create the genotoxic pressure under which DNA-damage resilience becomes clinically relevant [6, 7, 18, 19, 75]. TMZ benefit is strongly shaped by MGMT promoter methylation and MGMT-mediated repair capacity, consistent with the central role of alkylation repair in treatment response [6, 7, 75]. Longitudinal studies show that alkylator exposure can select for MSH6 alteration or mismatch-repair loss in a subset of tumors [8, 9, 81–83], promote TMZ-associated hypermutation in some recurrences [81, 82], and generate divergent molecular trajectories, including branched evolution at recurrence [81, 82].

The best-supported chemokine-linked DNA-damage response pathway in glioblastoma is the pericyte-derived CCL5–CCR5 axis [29]. Pericytes secrete CCL5, which activates CCR5 on adjacent glioblastoma cells and triggers an AKT–DNA-PKcs DNA-damage response module after TMZ exposure [29]. Functionally, this paracrine circuit reduces the lethality of alkylating injury by promoting repair signaling and limiting DNA-damage accumulation [29]. In preclinical glioblastoma models, pericyte depletion or disruption of CCL5–CCR5 signaling, including CCR5 blockade with maraviroc, increased DNA damage, attenuated repair signaling, enhanced TMZ cytotoxicity, and prolonged survival [29]. CXCL12–CXCR4 should be framed more narrowly as a damage-buffering niche rather than as a direct DNA-repair pathway [27, 67, 78, 86, 87]. After radiotherapy, hypoxia and vascular injury induce CXCL12-dependent recruitment of CXCR4-positive bone-marrow-derived myeloid cells that support post-irradiation vasculogenesis [27, 87, 90, 91]. In the SVZ-homing GB138 xenograft model, CXCL12 promotes mesenchymal-like activation and radioresistance [67], while TMZ-resistant models connect CXCL12–CXCR4 signaling to FOXM1-associated survival programs [86].

**Fig. 2:**
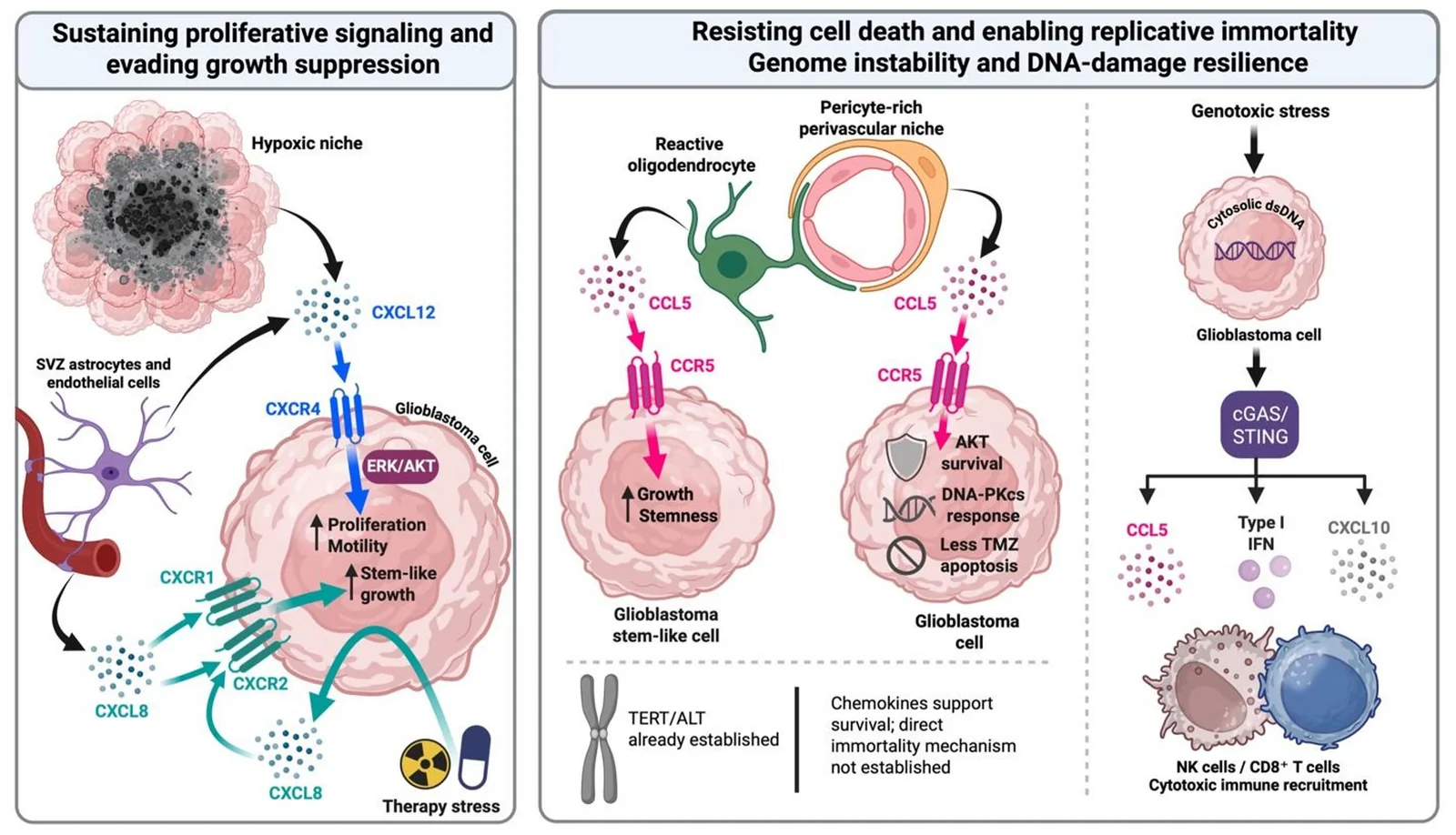
Chemokine-assisted growth, survival, and DNA-damage resilience. CXCL12–CXCR4 and CXCL8 signaling through CXCR1 and CXCR2 support proliferative, migratory, and stem-like states in glioblastoma. Stromal CCL5–CCR5 signaling promotes tumor-cell survival and temozolomide resistance through AKT–DNA-PKcs signaling, whereas genotoxic stress can activate cGAS–STING signaling and induce inflammatory chemokines that recruit immune effector cells. ALT, alternative lengthening of telomeres; IFN, interferon; NK, natural killer; STING, stimulator of interferon genes; TERT, telomerase reverse transcriptase; TMZ, temozolomide

Chemokines can also emerge downstream of unresolved DNA damage [88, 89, 92–95]. STING activation exemplifies this link by inducing type I interferon together with CXCL10 and CCL5. In glioma models, this response is associated with enhanced T-cell recruitment, while newer STING-agonist approaches show increased CD8⁺ T-cell and NK-cell effector responses and prolonged survival in preclinical models [88, 92, 93] and are advancing into clinical trials. Conversely, targeting DNA-repair pathways may disrupt chemokine-supported treatment resistance even when the therapeutic target is not a chemokine receptor. The DNA-PK inhibitor peposertib has been evaluated in a phase I study with radiotherapy followed by TMZ in newly diagnosed MGMT-unmethylated glioblastoma (NCT04555577) [96]. This is mechanistically relevant because DNA-PK functions downstream of pericyte-derived CCL5–CCR5 signaling and independently promotes TMZ resistance in recurrent glioblastoma models [29, 97]. However, whether DNA-PK inhibition disrupts this perivascular chemokine circuit or converts chemokine-supported repair into tumor-cell elimination remains unproven clinically [29, 96]. The principal chemokine circuits linking growth, survival, and DNA-damage resilience are summarized in Fig. 2.

### Inducing angiogenesis and vascular adaptation

Microvascular proliferation and necrosis are defining histopathologic features of glioblastoma [12, 13], and they are accompanied by vascular permeability, edema, hypoxia, and regional perfusion failure [13, 98–101]. In this setting, chemokines are best understood not as substitutes for VEGF, but as regulators of vascular adaptation - especially after hypoxia, radiotherapy, or anti-VEGF pressure [27, 78, 102–105]. ELR-positive CXC chemokines - including CXCL1, CXCL2, CXCL5, and CXCL8 - contain an N-terminal glutamic acid–leucine–arginine motif and are generally pro-angiogenic [102–104], often acting through CXCR2 on endothelial and myeloid compartments [103, 104, 106, 107].

CXCL12–CXCR4 is the best-supported chemokine circuit in post-radiotherapy vascular repair. In orthotopic glioblastoma xenografts, irradiation-induced vascular damage increased tumor hypoxia and HIF-1 activity, promoting CXCL12-dependent recruitment of CXCR4⁺ bone-marrow-derived CD11b⁺ myelomonocytic cells. These cells localized predominantly around rather than within the endothelium and supported vascular remodeling and restoration of tumor perfusion, thereby enabling regrowth of surviving tumor cells; blocking CXCL12–CXCR4 recruitment impaired vascular recovery and delayed or prevented recurrence [27].

Early clinical studies support feasibility but not mechanism-specific efficacy. In MERT, continuous plerixafor initiated near the completion of concurrent chemoradiation produced perfusion-MRI and histopathologic changes compatible with reduced postirradiation revascularization [78]. GLORIA initially combined radiotherapy with the CXCL12-neutralizing aptamer olaptesed pegol in incompletely resected or biopsied, MGMT-promoter-unmethylated glioblastoma; a six-patient expansion added bevacizumab and broadened eligibility to permit fully resected tumors [108, 109]. Neither study established spatial intratumoral CXCL12 suppression or a randomized survival benefit; the perfusion changes and post-hoc survival signals therefore remain hypothesis-generating.

Other vascular chemokine circuits are less therapeutically developed. ACKR3 can scavenge extracellular CXCL12 and thereby regulate its availability and CXCR4 signaling, although its contribution in GBM remains incompletely defined. By contrast, CCL2-related vascular effects appear largely indirect, through recruitment of macrophages that provide VEGF, matrix-remodeling enzymes, and trophic signals [21, 84, 85, 110–113]. Thus, the strongest therapeutic rationale in this hallmark is temporally targeted interruption of post-treatment vascular repair rather than generalized chemokine blockade.

### Activating invasion and dissemination

Glioblastoma cells extend beyond contrast-enhancing disease along white-matter tracts, including the *corpus callosum*, and along perivascular, subpial, and periventricular routes [114–127]. Across published cohorts, reported enhancing progression remains predominantly local/in-field after upfront chemoradiation (~ 80%) and salvage therapy (~ 60%) among evaluable cases, highlighting the distinction between infiltrative biology and radiographically apparent spatial failure [128]. Chemokines contribute chiefly as spatial guidance and niche-permissive signals. CXCL12-rich hypoxic, vascular, and SVZ regions attract CXCR4-positive invasive or stem-like tumor cells; interstitial flow may reinforce this chemotaxis [23, 24, 55–60, 62, 129, 130]. Endothelial-derived or necrosis-associated CXCL8 couples signaling through CXCR1 and CXCR2 to stem-like maintenance and motility [22, 131–133]. Microglia/macrophage-derived CCL5 promotes CCL5–CCR5-dependent invasion through Ca²⁺/CaMKII regulation of MMP2, whereas tumor-associated-macrophage-derived CCL8 promotes invasion and stem-like traits through CCR1 and CCR5 and ERK1/2 signaling [25, 134, 135]. Maraviroc attenuates CCR5-dependent glioblastoma invasion preclinically, but no chemokine circuit has been clinically validated for this purpose [134]. The observation that CX3CL1–CX3CR1 can restrain invasion in some models further underscores dependence on ligand source, receptor distribution, and anatomical context [136].

Clinical evaluation of invasion remains difficult because diffuse infiltration is established at diagnosis and is poorly captured by conventional radiographic criteria [123, 137–139]. The failure of the non-chemokine integrin inhibitor cilengitide to improve survival illustrates that preclinical anti-invasive activity does not readily translate into clinical benefit [140–143]. The Response Assessment in Neuro-Oncology 2.0 (RANO 2.0) criteria largely exclude nonenhancing disease from response assessment in IDH-wildtype glioblastoma, except after antiangiogenic therapy, further limiting direct pharmacodynamic evaluation of invasion [139].

### Tumor-promoting inflammation and immune evasion

Glioblastoma is a myeloid-enriched disease [40, 144–148]. The central immune problem is not merely leukocyte absence, but the organization of infiltrating immune cells into tissue-repair programs that support immunosuppression, vascular remodeling, and tumor persistence rather than durable cytotoxic control [31, 40, 146–149]. Chemokines are central to this architecture because they determine which immune cells enter the tumor, where they localize, and which tumor-supportive niches they reinforce [145–147, 149–157]. The relevant issue is which chemokine circuits recruit and position suppressive monocytes, MDSC-like cells, macrophages, and regulatory T cells [21, 158–162]. A complementary question is why cytotoxic T and NK cells fail to enter the tumor and sustain effector function.

Human single-cell and spatial studies now provide the framework for interpreting this biology [26, 40, 150–156, 163]. Glioblastoma immune infiltrates are dominated by heterogeneous myeloid populations comprising tissue-resident microglia and recruited monocyte-derived macrophages, whose relative abundance and functional states vary with ontogeny, hypoxia, tumor region, disease stage, and treatment exposure [26, 31, 40, 144–149, 156, 157, 163]. These studies also explain why leukocyte abundance alone is an insufficient biomarker [146–149, 153, 156]. A tumor with abundant immune cells may remain profoundly immunosuppressive if those cells are CCR2-positive monocytes, monocytic MDSC-like cells, repair-like macrophages, or Tregs rather than activated cytotoxic lymphocytes [31, 40, 146–148, 157]. Spatial context is equally important: hypoxic, perivascular, necrotic, and therapy-injured regions can each generate distinct chemokine gradients that recruit, retain, and reprogram immune populations [26, 40, 150–156, 163]. Spatial analyses indicate that effector T cells in primary glioblastoma are often sparse and concentrated in perivascular cuffs, with limited penetration into the tumor parenchyma [164–167].

Across spatially diverse immune niches, the most consistent chemokine function is selective recruitment and positioning of suppressive myeloid populations rather than generalized inflammation. In the orthotopic immunocompetent KR158B model, glioma cells secreted CCL2 and CCL7, which acted redundantly through CCR2 to attract CCR2-positive/CX3CR1-positive suppressive myeloid cells; combined ligand neutralization was required to abolish chemotaxis [168]. CCR2 blockade alone or combined inhibition of CCR2 and CCR5 reduces or remodels suppressive myeloid infiltration and improves anti-PD-1 activity in murine glioma [158, 159]. In an immunocompetent CXCL8-humanized murine glioma model, CXCL8 produced by tumor cells, CD4-positive T cells, and myeloid cells promoted MDSC infiltration; CXCL8 neutralization or inhibition of CXCR1 and CXCR2 potentiated anti-PD-1 therapy [160]. Collectively, these data support chemokine inhibition as an immune-remodeling partner for checkpoint blockade, not as a stand-alone immune activator. A separate CCL2 pathway recruits regulatory T cells. In the orthotopic immunocompetent GL261 model, CCL2 produced by tumor-associated macrophages and microglia recruited CCR4-expressing FOXP3-positive Tregs [21]. Mixed-bone-marrow chimeras showed that CCR4-deficient FOXP3-positive Tregs were selectively impaired in brain-tumor accumulation, supporting direct trafficking of an established Treg compartment [21]. These experiments did not lineage-trace conventional CD4-positive T cells and therefore do not exclude peripheral conversion. However, lineage-oriented studies in orthotopic and autochthonous murine glioma, supported by human glioblastoma phenotyping, found thymus-derived Tregs to predominate [169].

Prior myeloid-targeting experience suggests that chemokine blockade alone is unlikely to generate durable antitumor immunity in glioblastoma [30, 170–179]. This limitation is illustrated by CSF-1R inhibition, which can reprogram tumor-associated macrophages and slow glioma progression in preclinical models [30], yet is limited by adaptive resistance and limited clinical monotherapy efficacy in glioblastoma [170, 171, 180]. Chemokine axes can regulate the recruitment, retention, and spatial positioning of suppressive myeloid cells and regulatory lymphocytes, but these circuits operate within a broader immunosuppressive ecosystem [21, 31, 40, 146–148, 170].

**Fig. 3:**
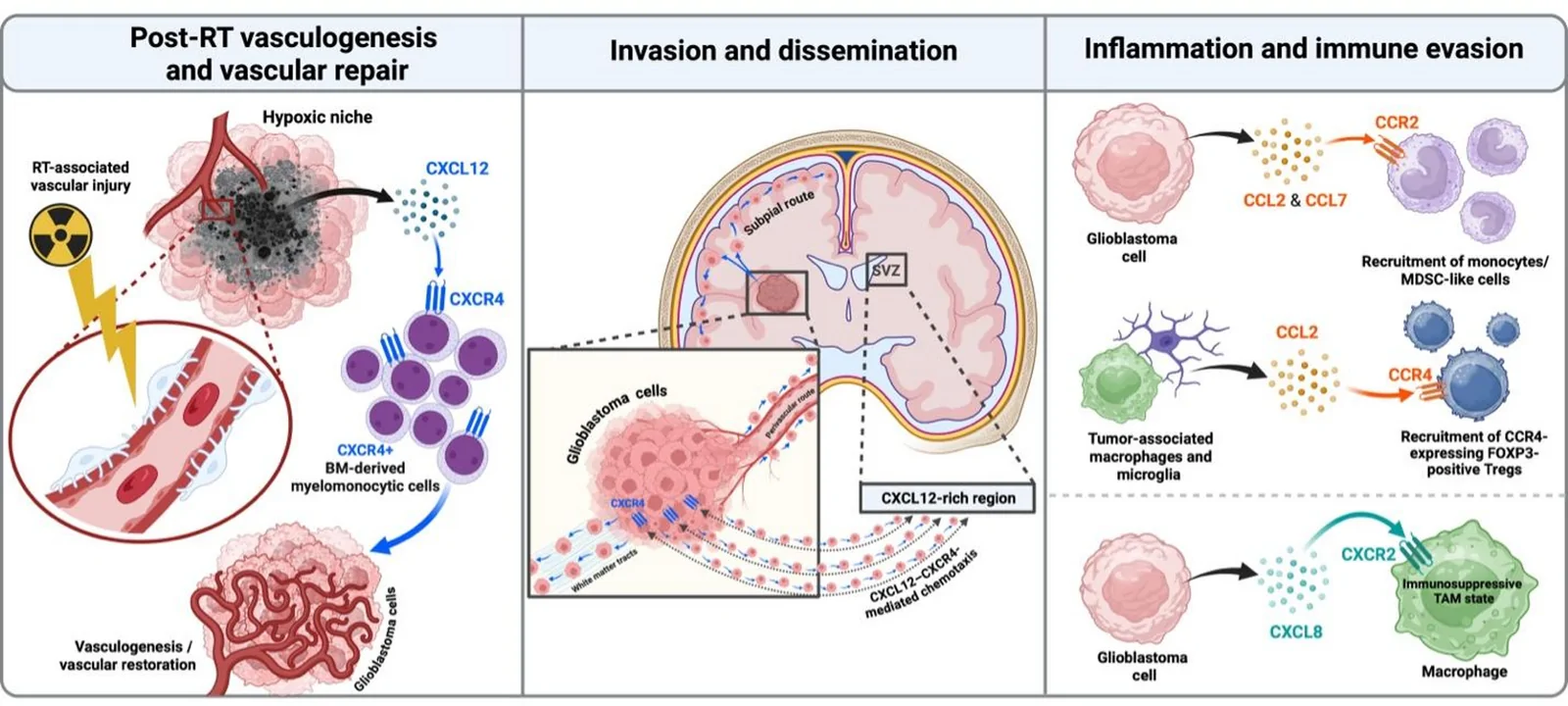
Chemokines in post-radiotherapy vascular repair, invasion, and immune evasion. CXCL12–CXCR4 promotes recruitment of bone-marrow-derived myelomonocytic cells that support post-radiotherapy vascular restoration and also guides glioblastoma-cell invasion within CXCL12-rich niches. Signaling by CCL2 and CCL7 through CCR2 recruits suppressive myeloid cells, CCL2–CCR4 promotes regulatory T-cell recruitment, and CXCL8–CXCR2 supports immunosuppressive macrophage states. BM, bone marrow; MDSC, myeloid-derived suppressor cell; RT, radiotherapy; Treg, regulatory T cell

Similarly, checkpoint blockade has not improved survival in unselected recurrent or newly diagnosed glioblastoma [172–174, 178]. However, neoadjuvant PD-1 blockade shows that tumor and systemic immunity can be pharmacologically perturbed, even when suppressive myeloid programs persist [175–177, 179, 181]. Thus, chemokine blockade should be viewed in the context of interventions that restore T-cell function, enhance antigen presentation, minimize corticosteroid exposure and treatment-associated lymphopenia, and increase tumor-cell vulnerability [157, 162, 182–187]. The major chemokine circuits linking vascular repair, invasion, and immune evasion are summarized in Fig. 3.

### Reprogramming cellular metabolism

Chemokine–metabolism interactions are best viewed as reciprocal multicellular circuits: metabolic states regulate chemokine production, while chemokine signaling reshapes cellular composition and metabolic programs. The clearest example is tumor-cell LDHA/lactate signaling, which activates ERK–YAP1/STAT3 and induces CCL2 and CCL7, thereby linking tumor glycolysis to CCR2-associated monocyte/macrophage recruitment [158, 188]. In turn, infiltrating macrophages release LDHA-containing extracellular vesicles that enhance glioblastoma-cell glycolysis, proliferation, and survival [188]. LDHA depletion or inhibition with stiripentol reduces macrophage infiltration and prolongs survival in preclinical models [188]. A complementary stromal circuit connects hypoxia-induced astrocyte-derived CCL20 to CCR6–NF-κB/HIF-1α adaptation in glioblastoma cells [189].

Lactate also produces compartment-specific epigenetic effects. In glioblastoma cells, CBX3–EP300-dependent histone lactylation increases CD47 expression and suppresses macrophage-mediated phagocytosis [190]. In monocyte-derived macrophages, PERK–ATF4-driven glucose metabolism promotes histone lactylation and IL-10 expression, thereby suppressing T-cell activity [191]. The CCL21–CCR7–HMGCS1 pathway also links paracrine chemokine signaling to tumor-cell-intrinsic lipid metabolism [192]. Lymphatic endothelial-like cells in glioblastoma secrete CCL21, which signals through CCR7 on glioblastoma stem-like cells. CCR7 activation induces KAT5-mediated HMGCS1K273 acetylation, stabilizing HMGCS1, a key enzyme in the mevalonate/cholesterol-biosynthesis pathway. This increased HMGCS1 stability enhances cholesterol synthesis and supports glioblastoma stem-like cell growth [192].

Clinical studies have not yet tested these chemokine-organized metabolic niches. Ketogenic-diet studies have primarily established feasibility and safety and did not evaluate chemokine-dependent pharmacodynamics [193, 194]. Translation will therefore require pathway-specific tissue readouts rather than metabolic intervention alone.

### Unlocking phenotypic plasticity and non-mutational epigenetic reprogramming

Phenotypic plasticity allows glioblastoma cells to shift among developmental-like malignant states (neural-progenitor-like, oligodendrocyte-progenitor-like, astrocyte-like, and mesenchymal-like) as well as hypoxic and therapy-tolerant programs, and such transitions can occur without acquisition of a new genetic driver [26, 39, 48, 195–197]. This hallmark is clinically important because recurrent glioblastoma is shaped not only by clonal selection, but also by therapy-associated state transitions, microenvironmental remodeling, and adaptive chromatin changes [26, 31, 80–82, 195–199]. Single-cell analyses show that glioblastoma tumor cells span multiple transcriptional states rather than fixed identities [26, 39, 48, 196, 197]. Defined transcriptional circuits can reconstruct tumor-propagating potential in glioblastoma stem-like cells [200]. Longitudinal multiomic studies further show that therapy reshapes the glioblastoma ecosystem through cell-intrinsic and cell-extrinsic mechanisms, with recurrent tumors often showing mesenchymal, inflammatory, and therapy-adapted chromatin programs [26, 31, 80–82, 195–199, 201].

CXCL8 signaling through CXCR1 and CXCR2 is best understood as a bidirectional niche circuit that couples tumor-cell plasticity to stromal and myeloid remodeling. Endothelial-derived CXCL8 maintains stem-like and invasive tumor states [22, 132], whereas TMZ-induced, tumor-cell-autocrine CXCL8–CXCR2 signaling alters histone programs and enriches tumor-initiating populations [28]. Mesenchymal glioblastoma stem-like cells also secrete CXCL8, which sustains GSC proliferation and self-renewal through PI3K–AKT and NF-κB signaling while driving immunosuppressive macrophage states through a paracrine CXCR2–JAK2–STAT3 pathway [31, 160, 202]. Thus, the same circuit can stabilize malignant-cell identity and remodel the surrounding immune compartment, particularly after treatment injury.

Direct blockade of CXCL8 signaling through CXCR1 and CXCR2 has not yet been clinically validated in glioblastoma. A distinct strategy instead exploits this axis for immune-cell trafficking: the recruiting phase I IMPACT study (NCT05353530) is evaluating CXCR2-modified CD70 CAR T cells in CD70-positive glioblastoma. This strategy exploits CXCL8 gradients to enhance immune-cell trafficking rather than blocking CXCL8 signaling in tumor cells [28, 160, 202]. CXCL12–CXCR4 is enriched in hypoxic and perivascular regions of glioblastoma and is linked to tumor-cell organization and Scherer secondary structures, particularly perivascular satellitosis [60]. Disrupting CXCL12–CXCR4 signaling impairs self-renewal and stem-like marker programs in rat RG2-derived glioblastoma stem-like cells [203], but no clinical study has specifically tested CXCL12–CXCR4 modulation using tumor-cell plasticity or stemness as the primary pharmacodynamic endpoint.

### Inducing or resisting senescence

During tumor evolution, malignant cells can bypass canonical replicative and oncogene-induced senescence through tumor-cell-intrinsic checkpoint, telomere-maintenance, and survival mechanisms [1, 3]. After radiotherapy or TMZ, senescence-like states have been demonstrated in malignant glioblastoma cells and non-malignant glia in several experimental systems, although their extent and persistence are model- and assay-dependent [204–210]. Among non-malignant cells, astrocytes have emerged as a prominent radiation-induced senescent population; single-nucleus profiling has also identified p16/p21-high oligodendrocyte progenitor cells, although the functional recurrence-promoting circuit was established for astrocytes [205, 209]. These metabolically active, apoptosis-resistant cells can acquire a senescence-associated secretory phenotype (SASP) enriched for cytokines, chemokines, growth factors, proteases, and extracellular-matrix-remodeling factors [204, 206–216].

In non-glioma systems, CXCL8 and CXCL1 can reinforce replicative and oncogene-induced senescence through CXCR2-dependent signaling [212], while IL-6/CXCL8 inflammatory networks can propagate oncogene-induced senescence programs [214]. Thus, senescence is contextual and may remain tumor-suppressive when immune surveillance clears senescent cells efficiently [217, 218], but becomes tumor-promoting when an unresolved SASP sustains chronic, repair-like inflammation [204, 208, 211–214].

**Fig. 4:**
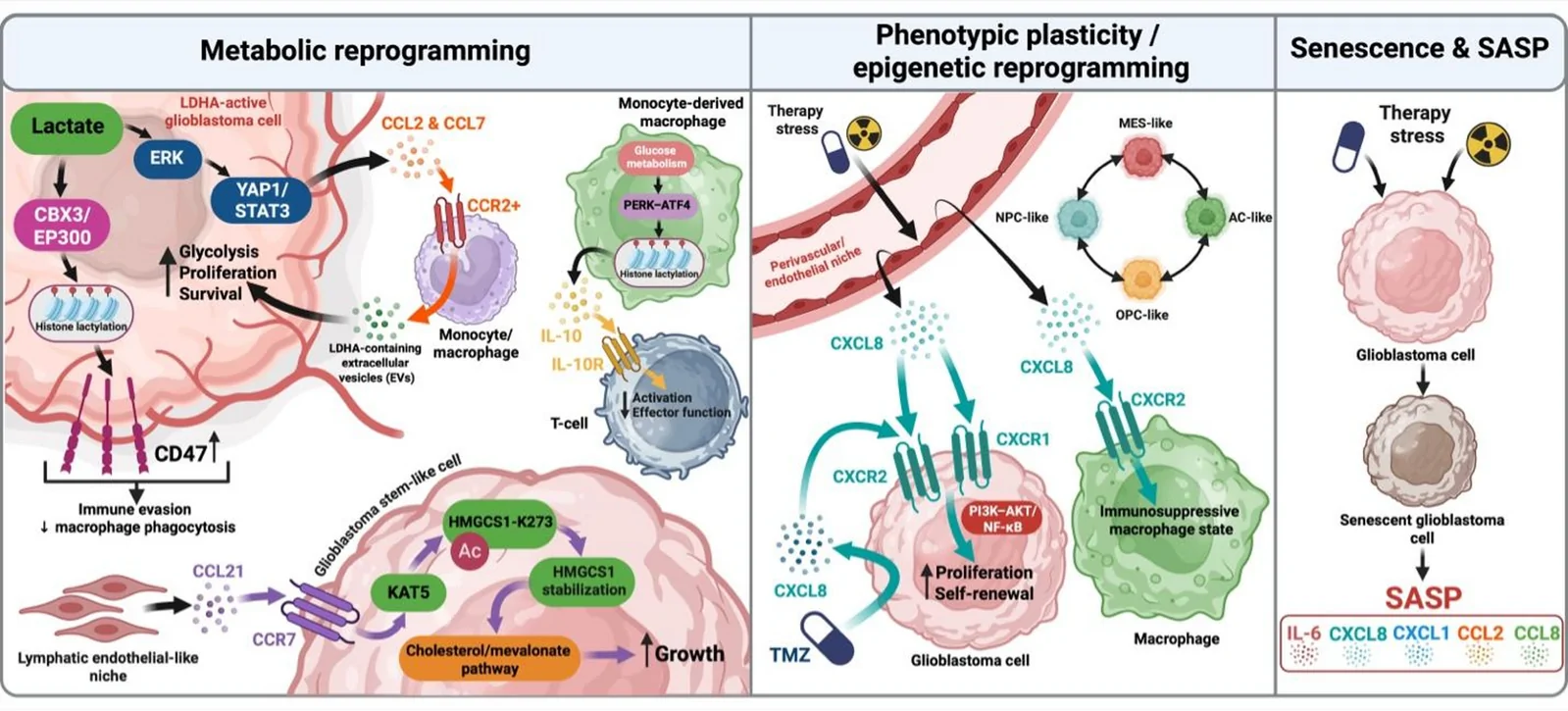
Chemokine–metabolism, plasticity, and senescence circuits. Metabolic and chemokine pathways form reciprocal multicellular circuits in glioblastoma. Tumor-cell LDHA/lactate signaling induces CCL2 and CCL7 and promotes myeloid recruitment, while macrophage-derived LDHA-containing extracellular vesicles enhance tumor-cell glycolysis and growth. Lactate-dependent histone modification and CCL21–CCR7-dependent cholesterol metabolism provide additional links between metabolic adaptation and immune or stem-like phenotypes. CXCL8 signaling through CXCR1 and CXCR2 supports therapy-associated tumor-cell plasticity and immunosuppressive macrophage states, whereas treatment-induced senescence generates a SASP containing multiple chemokines with context-dependent effects. AC-like, astrocyte-like; EV, extracellular vesicle; LDHA, lactate dehydrogenase A; MES-like, mesenchymal-like; NPC-like, neural-progenitor-like; OPC-like, oligodendrocyte-progenitor-like; SASP, senescence-associated secretory phenotype; TMZ, temozolomide

**Fig. 5:**
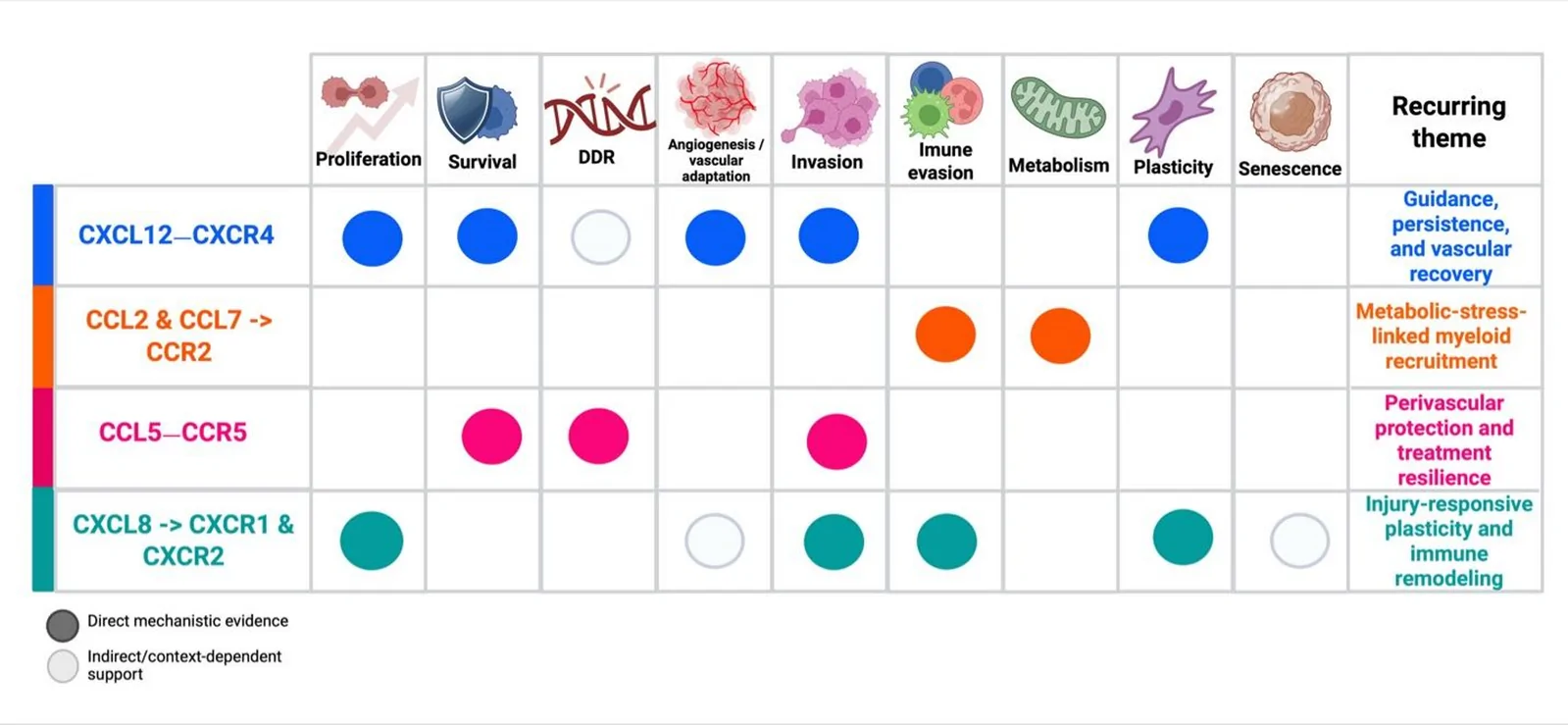
Recurrent chemokine circuits across glioblastoma hallmarks. The matrix summarizes four recurrent chemokine circuits and their principal associations across glioblastoma hallmarks. Filled circles indicate direct mechanistic evidence, open circles indicate indirect or context-dependent support, and blank cells indicate insufficient evidence for assignment. Assignments reflect the evidence discussed in the corresponding sections of the review. DDR, DNA-damage response

Treatment-induced senescence becomes relevant to chemokine biology when persistent SASP-producing cells escape immune clearance. In MGMT-low, mismatch-repair-competent models, TMZ induces O⁶-methylguanine-dependent DNA damage, MRN–ATR–CHK1 signaling and p53/p21-dependent senescence, together with an NF-κB-linked SASP containing CXCL8, CCL2, CCL8, and CXCL1 [204, 206, 215]. Recurrent human glioblastomas contain compatible DNA-damage, cell-cycle-arrest, and chromatin features, although these signatures do not by themselves prove stable senescence [206, 208, 215]. After radiotherapy, senescent astrocytes released a SASP that included CXCL12 and TNF-α; astrocyte-derived TNF-α activated Myc–Max signaling in glioblastoma cells, induced tumor-cell CXCL1, and recruited CXCR2-positive inflammatory myeloid cells [205]. Separately, depletion of p16INK4a-high malignant senescent cells in a non-treatment-induced mouse glioblastoma model remodeled the tumor ecosystem and prolonged survival [208]. Together, these findings provide a preclinical rationale for temporally sequenced senotherapy after cytotoxic treatment to limit persistent SASP-driven remodeling of the recurrence niche [205, 206, 208–210, 213, 215, 216, 219]. These ligands should not be treated as a single pathway. CCL2 can recruit CCR2-positive myeloid cells and CCR4-positive Tregs, whereas CCL8 can signal through CCR1 and CCR5 on glioblastoma cells [21, 135]. Whether therapy-induced senescent cells engage these receptor-defined circuits in vivo remains unresolved. The metabolic, plasticity, and senescence-associated circuits discussed above are integrated in Fig. 4, whereas recurrent chemokine circuits spanning multiple hallmarks are summarized in Fig. 5.

## Conclusions

Chemokine signaling in glioblastoma is best understood as a spatial and stress-responsive regulatory layer rather than as a primary oncogenic driver. Canonical genetic alterations establish the core malignant program, whereas chemokine circuits help determine how malignant, vascular, stromal, and immune cells are recruited, positioned, and functionally organized within hypoxic, perivascular, invasive, immunosuppressive, metabolically constrained, and treatment-injured microenvironments. The hallmarks framework is therefore useful not because individual chemokines correspond to individual hallmarks, but because it reveals recurrent circuits that connect multiple malignant phenotypes. The most coherent glioblastoma-relevant modules are CXCL12–CXCR4 in vascular repair, invasion, and stem-like persistence; CCL2 and CCL7 signaling through CCR2 in suppressive myeloid recruitment and metabolism-linked macrophage remodeling; CCL5–CCR5 in perivascular protection, invasion, and DNA-damage tolerance; and CXCL8 signaling through CXCR1 and CXCR2 in angiogenesis, myeloid suppression, and therapy-induced plasticity.

The therapeutic implication is not broad chemokine blockade or chemokine-directed monotherapy. Instead, translational development should focus on temporally defined, biomarker-selected dependencies arising under specific treatment pressures - for example, interrupting CXCL12-dependent vascular restoration after radiotherapy, suppressing CCR2- or CCR5-linked myeloid and repair programs, or targeting CXCR2-dependent plasticity after treatment injury. CXCL12–CXCR4 targeting has reached early clinical evaluation and produced pharmacodynamic signals, but durable survival benefit, spatially resolved intratumoral target engagement, and pathway suppression remain unproven.

Chemokine gradients may also be engineered or exploited to improve cellular immunotherapy, but ligand expression alone should not be interpreted as evidence of effective trafficking. Validation should include functional receptor expression and chemotactic responsiveness of the therapeutic cells, transendothelial migration, vessel-to-parenchyma penetration, regional cellular distribution, and cytotoxic activity throughout the tumor. Where feasible, future studies should incorporate paired pretreatment and on-treatment specimens with spatially resolved pharmacodynamic measurements. Functional endpoints should test whether an intervention reduces post-treatment vascular restoration, suppressive myeloid states, invasion, plasticity, or DNA-damage tolerance - and whether these changes delay recurrence or improve survival. Until these criteria are met, chemokine circuits should be considered context-dependent regulators and candidate therapeutic dependencies—not validated standalone therapeutic targets or established primary oncogenic drivers.

## Data Availability

No datasets were generated or analysed during the current study.
